# Fiber metal laminates for high strain rate applications with layerwise shock impedance tuning

**DOI:** 10.1038/s41598-023-45795-9

**Published:** 2023-10-27

**Authors:** Anand Pai, Marcos Rodriguez-Millan, Chandrakant R. Kini, Ravindra Mallya, Chandrakantha Bekal, Suhas Yeshwant Nayak, Satish B. Shenoy

**Affiliations:** 1 Department of Aeronautical and Automobile Engineering, Manipal Institute of Technology, Manipal Academy of Higher Education, Manipal, Karnataka 576104 India; 2 Department of Mechanical Engineering, University Carlos III of Madrid, Avda. de la Universidad 30, 28911 Leganés, Madrid Spain; 3 Department of Mechanical and Industrial Engineering, Manipal Institute of Technology, Manipal Academy of Higher Education, Manipal, Karnataka 576104 India

**Keywords:** Engineering, Materials science, Mathematics and computing

## Abstract

Novel materials such as fiber-metal laminates (FMLs) have demonstrated significant potential in a variety of applications. They must contend with problems such fatigue, creep, high-speed projectile impact, and deformation at high strain rates while in use. When employed as structural materials in aircraft, especially when exposed to shock wave impact and high velocity impact, fiber-metal laminates’ high strain rate characteristics become crucial. Shock impedance matching is a revolutionary approach used for shock-tuning the separate layers. The novelty of the current work is in developing custom shielding laminates, with in-depth analysis on the response of the shock impedance tuning of individual layers on the laminate behaviour at high strain rates. In the current study, five stackups of FMLs comprising metallic (AA 6061-T6) and fiber-reinforced polymer (FRP) plies, were formulated, incorporating shock impedance matching. The fiber-polymer plies used in the FMLs include ultra-high molecular weight polyethylene (UHMWPE), p-aramid for supplementing the impact resistance. Transmission loss functions (*TL*) estimated from the impedance tube experiments were used to indicate the shock tuning of the various laminates. The laminates underwent testing using a Split Hopkinson Pressure Bar (SHPB) apparatus to determine their properties at high strain rates ($350\, \textrm{s}^{-1}$ to $460\, \textrm{s}^{-1}$). The variation in the Shock Energy (*SE*) absorbed by the laminates at various strain rates was analyzed as a function of the corresponding Transmission Loss employing regression. The dynamic stress-strain curves showed an increase in shock energy absorption at higher strain rates. The sequence SSP-IV and SSP-II showed the highest values of energy absorption as well as Transmission Loss.

## Introduction

Fiber-metal laminates (FMLs) comprising alternately layered metallic with fiber-reinforced polymer laminae are found in several applications like aerospace, automotive, buildings, and shielding structures. Commonly utilized fiber-metal laminates include ARALL, CARALL and GLARE with superior impact resistance and fatigue performance^1–3^. During the regular operation, the structures may be subjected to spontaneous impact events like crash or collison in the case of aircrafts and automobiles^4–6^, while buildings may be subjected to seismic activities like earthquakes^7^. Bird strikes pose a significant threat to aircraft, causing damage to the fuselage and endangering passengers^8^. FMLs with shock impedance grading can provide better protection by dispersing and absorbing the energy from bird impacts. Further research and development in this area can lead to the creation of FMLs with optimized material compositions and microstructural designs to enhance their impact resistance capabilities^9^. The aerospace industry is continuously looking for materials and structures that can survive high-velocity impacts, such as those brought on by damage from debris or foreign objects^10^. In order to protect crucial components’ structural integrity, FMLs with high strain rate characterisation can be designed to endure severe shock effects. FMLs offer the potential for increased resilience and safety in aircraft structures. By incorporating advanced materials and innovative designs, FMLs can exhibit superior damage tolerance and fracture toughness. This resilience can enable the aircraft to withstand and safely absorb impact loads, reducing the risk of catastrophic failures and enhancing passenger safety^11^. Shielding structures designed for protection against ballistic, shockwave and blastwave impact suffer enormous material deformation in negligible time^12,13^. One of the shielding applications include whipple shields which protect the satellites and spacecrafts against hyper-velocity impact^14^. Thus, the mechanical response of such structures under dynamic loading conditions and high strain rates is vital from the aspect of engineering design. The high strain rates for the ballistic and blast impacts reach >($10--10^4\, \textrm{s}^{-1}$)^15–19^.

Typical construction of fiber-metal laminates include metallic/ alloy skins stacked alongside fiber reinforced polymer laminae^6,20–23^. In many of the works, one fiber ply type and one metallic ply type^24–26^ have been used, with the order of the arrangement being arbitrary. When higher number of fiber ply types are included, the response of the fiber-metal laminates is significantly affected by the order of the plies^27,28^. In one of our recent works^29^, in order to identify the ordered arrangement with the maximum Transmission Loss, shock impedance matching of fiber-metal laminates has been carefully examined using computational, analytical, and experimental methods. In another of our recent works^30^, the sequences have been subjected to shockwave impact experiments using a shocktube. Although this study provided useful information on the deformation profiles and ply failure modes, the mechanical performance at high strain rates could provide further insight on the capability and quality of the stacking sequences.

High rate of strain characterisation ($10^2--10^4\, \textrm{s}^{-1}$) commonly uses the Split Hopkinson Pressure Bar (SHPB). Developed by Kolsky^15^, the apparatus saw several enhancements to cater to a broader class of materials—metals, concrete, adhesives, composites^31–33^. Grote et al^34^ have employed a SHPB made up of a set of 12.7 mm diameter steel bars with a yield strength of *sim* 1800 MPa (the striker bar, incident pressure bar, transmitter bar, and momentum trap). The loading pulse is transferred to the pressure bar in the form of a compression wave by the axial impact when the striker bar strikes the incident pressure bar with a high impact velocity. The specimen is deformed by the compression wave with a pressure pulse whose constant amplitude and duration are proportional to the striker bar’s length. The impact velocity may be regulated by varying the air gun’s pressure since the amplitude of the incident pulse is inversely related to the impact velocity. SHPB and Direct impact tests have been used in tandem for several classes of materials^35,36^. Richter et al^37^ deployed Digital Image Correlation (DIC), a non-contact technique, to track the strains during the SHPB’s high-speed strain testing. Yang et al.^38^ explored the behaviour of aramid fibre-reinforced polymer (AFRP) confined concrete subjected to high strain-rate compression at strain rates ranging from 80 to 170 s$^{-1}$. Kevlar CAS-415 AFRP was used to wrap cylindrical concrete samples with epoxy resin as the binder. Single wrap, twin wrap and three-layer wrap samples along with bare concrete samples were tested at different strain rates. Twin ply AFRP wrapped concrete performed better than the other samples with better ability to redistribute the internal forces coupled with the viscoelastic character of the hardened cement paste and time-dependent micro-cracks growth. Additionally, the twin AFRP ply wrapped concrete showed similar ultimate strain values $\sim$0.033) at the different strain rates. The uncovered concrete showed disparities in its response to different strain rates. The dynamic increase factor (DIF) and the logarithmic strain rate were discovered to have an operational relationship by the authors. Gardner et al.^39^ determined the dynamic constitutive properties of sandwich constructions built of E-glass vinyl ester facesheets using a SHPB device with a hollow transmission bar. The sandwich panels comprised $Corecell^{\textrm{TM}}$ A-series foam with a polyurea interlayer. The core consisted of three layers of A-series foams with increasing density with a polyurea interlayer. Two configurations with the polyurea layer placed before the lightest foam, and the other where polyurea was placed after the heaviest foam were developed. The overall thicknesses of the sandwich panels were kept constant at 4.8 mm. The foams displayed an augmented response to higher strain rates able to absorb higher energies. Sassi et al .^40^ have studied how adhesively bonded glass fiber-polyester laminates behave in dynamic compression utilising hopkinson bars and the impact of high strain rates. The bonded laminates displayed high strain rate sensitivity, the authors discovered, with brittle fractures at the polyvinylester adhesive interfaces. Li et al.^41^ studied the influence of high strain rates (up to 2051 s$^{-1}$) on 3-D braided composites employing split hopkinson devices under dynamic compression on braids with various braiding angles. The dynamic characteristics improved as the strain rate increased, while the strain to failure decreased. Shear fracture, fibre breaking, interface debonding, and matrix cracking were the composites’ failure modes. The amount of dynamic damage and fracture decreased when the braiding angle was raised. Sharma et al.^42^ analysed the glass fibre reinforced epoxy-AA2024 laminates’ high strain rate response under tension using a split Hopkinson pressure bar rig. The strain rate was calculated using a DIC method. The tensile strength significantly increased at high strain rates, according to the authors. Studies on the dynamic characterisation of fibre metal laminates have frequently used split Hopkinson Pressure bar tests^43–45^. Khan and Sharma^45^ assessed the high strain rate response (400–480/s) of FMLs made of glass fiber plies and AA2024-T3 layers using Split Hopkinson bar. The rate sensitivity influences the matrix cracking and delamination among the layers. In another study on glass-fiber/ AA2024-T3 FMLs, Sharma et al.^46^ used a split hopkinson bar along with digital image correlation setup for strain measurements for high strain rate response. The authors observed that highest strength was observed in the FML with all the glass-fiber layers stacked together, possibly attributed to fiber bridging. Zarezadeh-mehrizi et al.^47^ modified the FMLs containing glass fiber epoxy/AA6061-T6 by inserting a natural rubber elastomeric layer. The recent works on split Hopkinson pressure bar characterization have been summarized in Table 1.

**Table 1:** Recent research on high strain rate characterization using split Hopkinson pressure bar.

Researchers	Material	Configuration	Strain rates (s$^{-1}$)	Characterization	Findings
Malinowski et al.^48^	Aluminium	Monolithic	10$^3$	Compression	Friction and inertial effects
Gardner et al.^39^	E-glass vinyl ester/corecell foams	Multi-layered	4800–5400	Compression	Hollow tranmission bar
Imbalzano et al.^49^	AA5083-H116	Multi-layered	10$^3$–10$^4$	Compression	Response to impulsive loading
Yang et al.^38^	Concrete/aramid reinforced epoxy	Multi-layered	80–170	Compression	Hybrid configurations better than plain concrete
Miao et al.^50^	Epoxy	Polymer	330	Compression	Vertical split Hopkinson bar
Sharma et al.^42^	Glass fiber reinforced epoxy-AA2024 laminates	Multi-layered	250	Tensile	Improvement in tensile properties
Sassi et al.^40^	Fiberglass reinforced polyester	Multi-layered	372–1030	Compression	Quality of adhesive assessed
Li et al.^41^	3-D braided carbon reinforced epoxy	Fiber reinforced composites	2051	Compression	Failure modes identified
Gao et al.^51^	A1070	Monolithic	10$^4$	Thermo-mechanical	Sensitivity of thermocouple and infrared detectors
Zhang et al.^52^	$\alpha$-Ti alloy	Monolithic	10$^3$–10$^6$	Thermo-mechanical	Temperature rise during high strain rates

**Figure 1:**
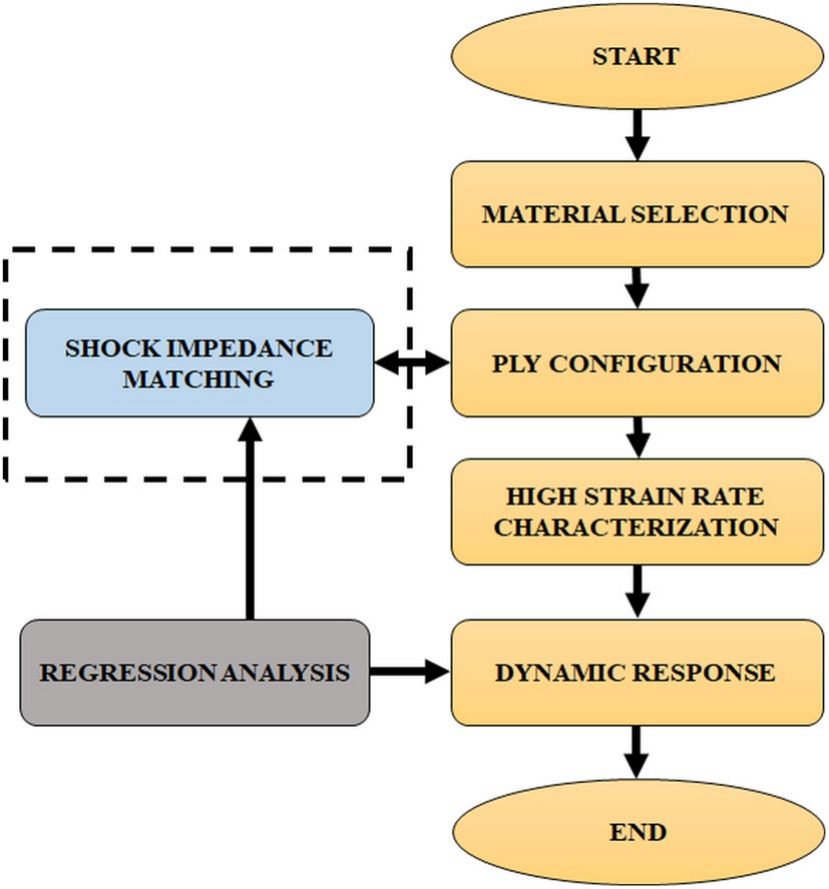
Methodology for studying the influence of shock impedance matching on high strain rate response of laminates.

**Figure 2:**
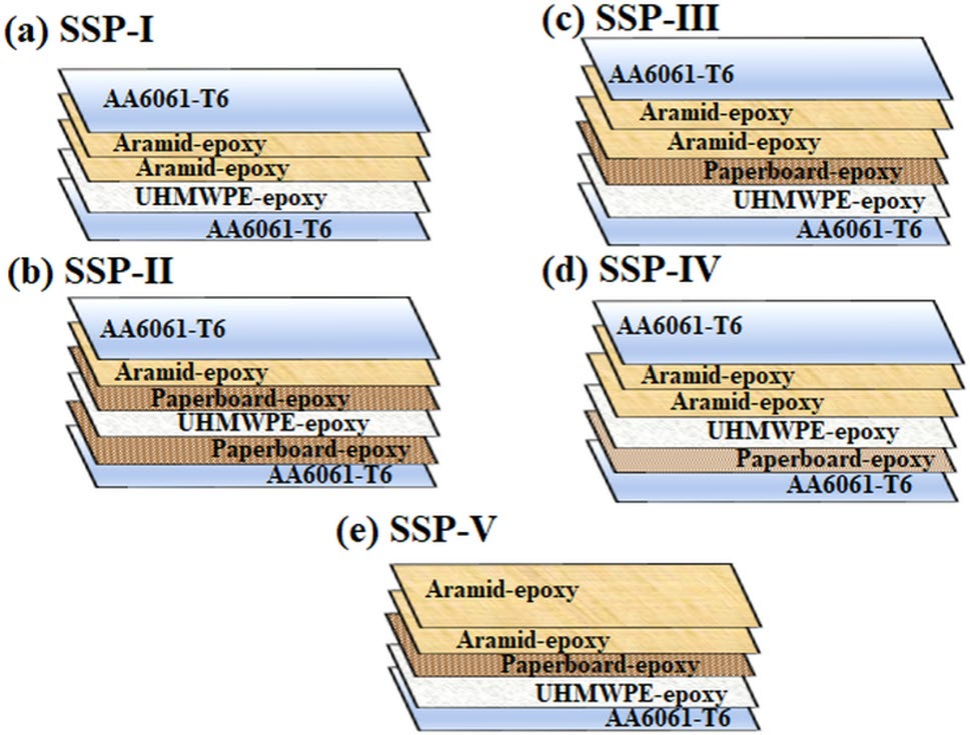
Representation of the layered arrangements (SSPs) with dimensions.

The novel aspect of the current work is the creation of specialized shielding laminates, together with a thorough analysis of the impact of shock impedance tuning for individual layers on laminate behavior at high strain rates. The FMLs comprised high-performance, ballistic grade fiber-reinforced plies, made of aramid and UHMWPE fabrics, along with a low shock impedance, partially auxetic sheet of paperboard. AA6061-T6 skins have been used to sandwich the fiber-reinforced plies in the FMLs. To incorporate the shock impedance matching, the order and the number of the core layers comprising aramid bi-directional layer, UHMWPE layer, and the paperboard layer was varied, with epoxy binder. Five configurations were considered for the study (refer Fig. 2) and each configuration was assigned a roman numeral succeeding the nomenclature SSP (Stratified Sandwiched Panels). The approach used in the study to determine how the plies’ shock impedance varied with the high strain rate response is shown in Fig. 1.

## Materials and methods

### Materials

The AA6061-T6 sheets of metallic skins, which are 0.7 mm thick, were provided by Hi-Tech Sales Corporation in Mangalore, India. The ballistic grade materials used in the laminates comprised the aramid BD 480 GSM, woven fabric (plain weave, yarn count balanced in warp and weft directions), the UHMWPE UD 130 GSM fabric, and epoxy resin (CT/E 556 epoxy resin and CT/AH 951 polyamine hardener) were all bought from Composites Tomorrow Inc. based at Gujarat, India. The 650 GSM paperboard sheets were purchased from Vijay Papers in Karnataka, India. Table 1 shows the shock impedance values and densities of the constituent materials. The provided epoxy resin had a pot life of 30 minutes, a density of 1150 kg/m$^3$, and a mix viscosity of 1500 mPas. Table 2 shows shock impedance values and densities of the constituent materials.

**Table 2:** Physical properties of the constituent materials^29,39^.

Role of the ply	Ply material	Mass density (kg/m$^3$)	Shock impedance $\times 10^6$ (Ns/m$^3$)
Facet skin plate	AA6061-T6	2750	15 ± 1.25
Core layers	Aramid-epoxy ply	1380	0.3 ± 0.08
	Paperboard-epoxy ply	1210	1.07 ± 0.21
	UHMWPE-epoxy ply	1190	4.51 ± 0.72
Distal skin plate	AA6061-T6	2750	15 ± 1.25

### Fabrication process

**Figure 3:**
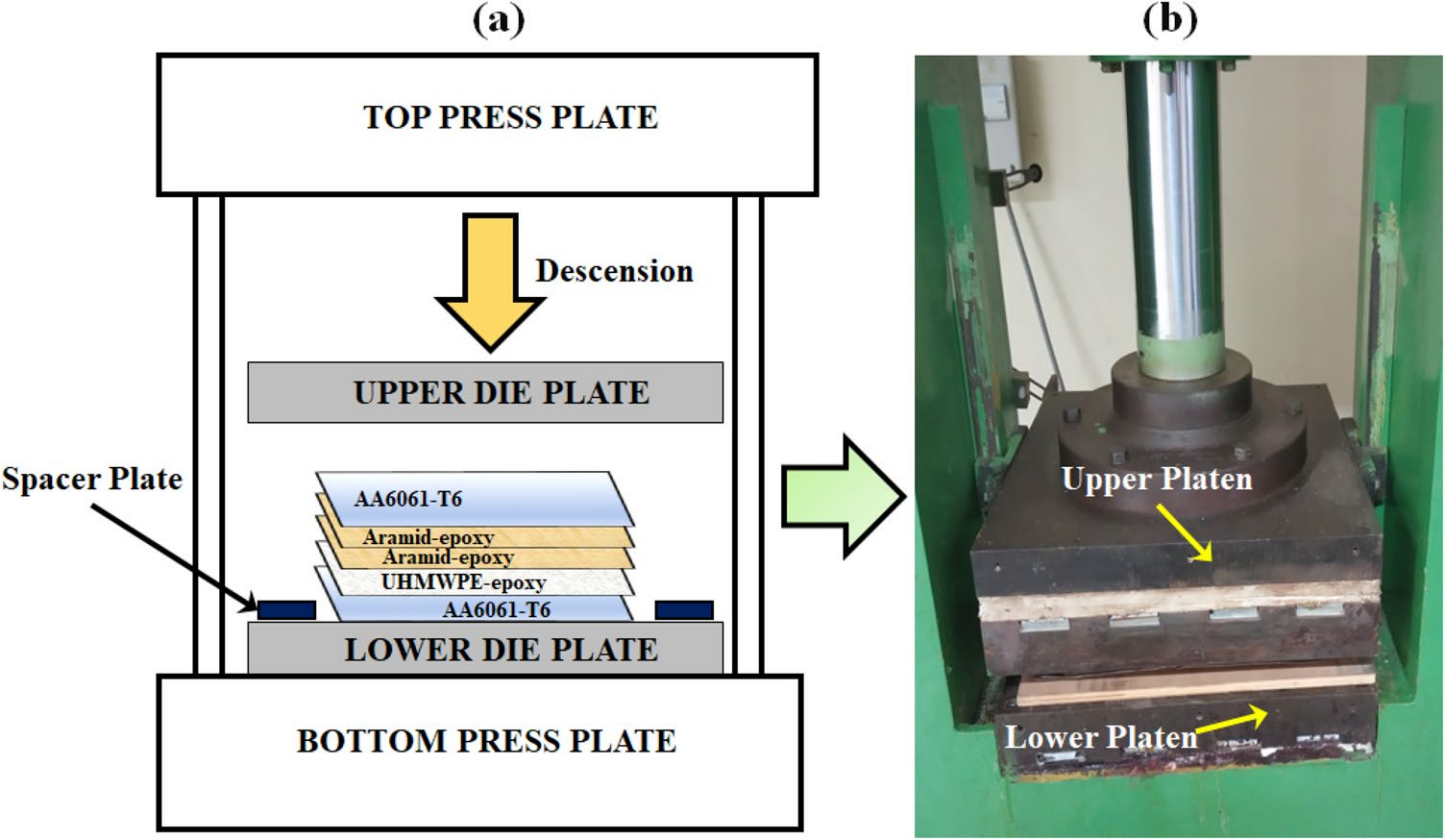
(**a**) Fabrication setup schematic (ex: SSP-I sequence). (**b**) Compression moulding machine.

One of the common methods for producing fibre metal laminates, compression moulding, was used to construct the various combinations^53,54^. The fabrication setup is shown in Fig. 3. To increase the interfacial adhesion, the aluminium alloy AA6061-T6 surfaces were sanded with grit papers^54^. The mold release agent was applied to the bottom die plate and a peel ply was placed above it. The different plies were then pre-processed, weighed for each stacking sequence, and progressively stacked on top of one another using the Hand Layup method. Between the layers, the premixed resin/hardener mixture (in the ratio 10:1 by weight) was uniformly coated as per the supplier recommended fiber-to-matrix weight fractions. The upper die-plate (coated with mold release agent) was then positioned above the peel ply, placed over the upward facing surface of the AA6061 layer (distal). Precisely machined spacers were placed between the upper and lower die plates for ensuring uniform thickness of the stackups. Each arrangement was transferred to the cold pressing machine, pressed, and held for a dwell period of $\sim$25 h, at room temperature to facilitate curing as per the supplier recommendations. After curing, laminates were sent out for water jet machining to create test specimens. For the experimentation on the impedance tube (circular specimens of 30 mm and 100 mm diameter) and SHPB (circular specimens of 10 mm diameter) were cut by water jet machining. After the water jet cutting, the specimens of all sequences were inspected; no delamination or debonding among the plies were noticed.

### Shock impedance matching

**Figure 4:**
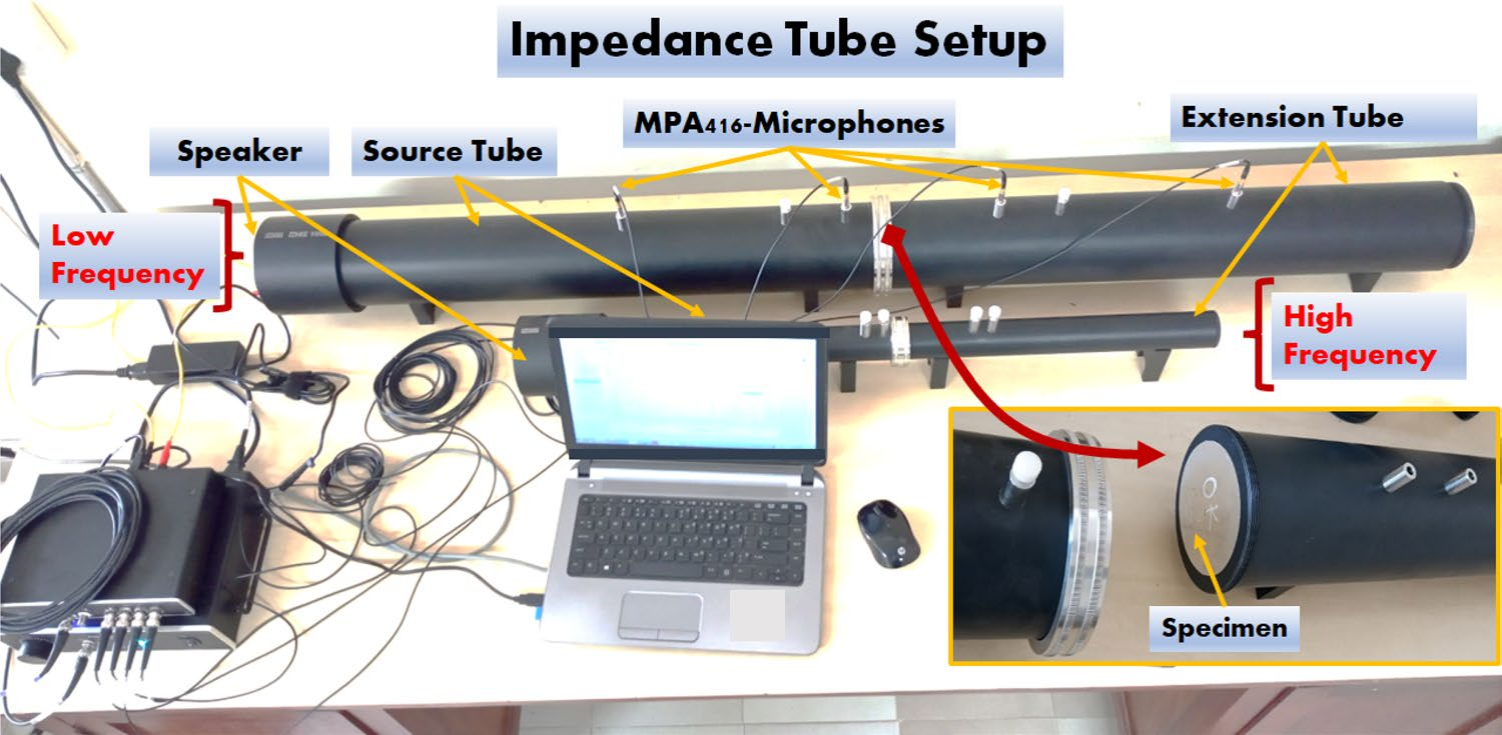
Impedance tube setup for transmission loss measurement.

Shock impedance grading affects the intensity of shock waves transmitted, demonstrating the efficacy of the shock shielding^55^. Transmission Loss measurements were made for each of the sequences in the frequency range of 0 to 6300 Hz using the impedance tube experiments on the various sequences. The impedance tube apparatus (make: BSWA SW) as shown in Fig. 4, comprises a high diameter setup- SW 30-L/SW 30-E (30 mm) for low frequency range (0–1500 Hz) and a low diameter setup-SW 100-L/SW 100-E (30 mm) for high frequency (1600–6300 Hz) for measuring the transmission losses. A four-microphone transfer function served as the basis for the measurement of Transmission Loss. Between the source tube and the extension tube, the specimen (of a particular sequence) was positioned within the holder. The loudspeaker was 4 in. in diameter, with power rating of 20 W, and resistance 8 $\sim {\omega }$, frequency range 20 Hz to 8 kHz. The speaker was turned on, and after 10 min, the microphone readings for the designated frequency range were recorded. The range of frequency distribution of sound transmission loss was obtained for all the stackups. The sound transmission loss distribution for the frequency range was acquired. For each sequence, two sets of specimens were subjected to the transmission loss measurements.

The key focus metrics from the study i.e. the transmission loss, denotes the amount of energy that each specimen fails to transmit, as shown in Eq. (1).

1 $$\begin{aligned} TL=10\log {\frac{I_{inc}}{I_{tran}}}=10\log {\frac{1}{\tau _c}} \end{aligned}$$

### Dynamic testing using split Hopkinson pressure bar

**Figure 5:**
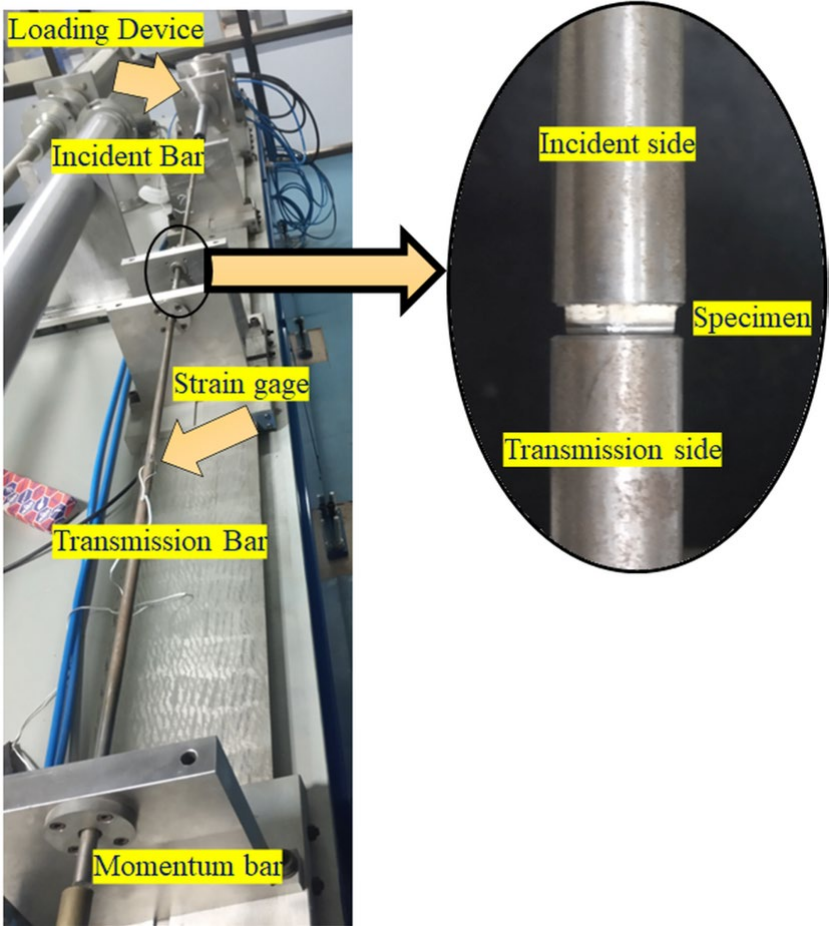
Experimental setup of the split Hopkinson pressure bar.

In the current SHPB setup, the striker bar, incident bar, transmitter bar and the momentum bar were made of maraging steel (elasticity modulus of 190 GPa and density $\sim$ 8000 kg/m$^3$). The experimental setup of the Split Hopkinson pressure bar is shown in Fig. 5.

When the loading device strikes the incident bar (time, $t=0$), a one-dimensional pressure wave travels in the direction of the specimen. At the free end, the compression wave is reflected as a tension wave. At the bar/specimen contact, this unloading wave is repeatedly reflected, with the remaining energy passing through the transmission bar. Strain gages were used to measure the stresses in the bars, and the data was sent to a data acquisition setup that includes a Wheatstone bridge for signal conditioning and a pre-amplifier to boost the voltage before it is sent to the oscilloscope.

2 $$\begin{aligned} c_B= & {} \sqrt{\frac{E_m}{\rho _{m}}} \end{aligned}$$

3 $$\begin{aligned} V_1= & {} c_B\left( \epsilon _I-\epsilon _R\right) \end{aligned}$$

4 $$\begin{aligned} V_2= & {} c_B\left( \epsilon _T\right) \end{aligned}$$

5 $$\begin{aligned} \dot{\epsilon }= & {} \frac{V_1-V_2}{L_s}=\frac{c_B\left( \epsilon _I-\epsilon _R-\epsilon _T\right) }{L_s} \end{aligned}$$

6 $$\begin{aligned} \epsilon _E= & {} \int _{0}^t\dot{\epsilon }dt=\frac{c_B}{L_s}\int _{0}^t\left( \epsilon _I-\epsilon _R-\epsilon _T\right) dt \end{aligned}$$

7 $$\begin{aligned} F_1= & {} A_BE_m\left( \epsilon _I+\epsilon _R\right) \end{aligned}$$

8 $$\begin{aligned} F_2= & {} A_BE_m\left( \epsilon _T\right) \end{aligned}$$

9 $$\begin{aligned} \sigma= & {} \frac{\left( F_1+F_2\right) }{2A_S}=\frac{A_BE_m}{2A_s}\left( \epsilon _I+\epsilon _R+\epsilon _T\right) \end{aligned}$$

**Figure 6:**
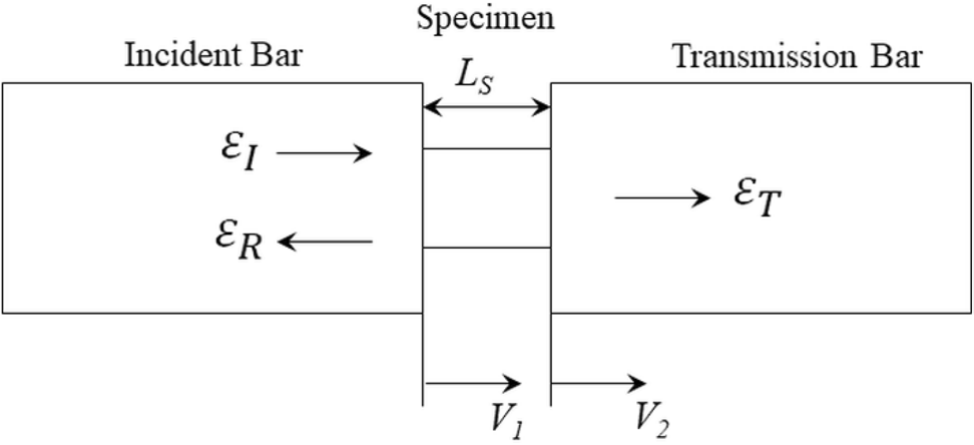
Test section of the split Hopkinson pressure bar^56^.

When the incident bar is struck at an impact velocity of ’$V_B$’, an incident elastic wave travels through the bar with a velocity of ’$c_B$’ which can be calculated from Eq. (2). ’$E_m$’ is the Young’s modulus of the elastic material of the bar and ’$\rho _{m}$’ is the mass density of the bar material. Figure 6 shows the test section of the bar setup. The velocities at the left face and right faces are $V_1$ and ’$V_2$’ respectively, which are given by Eq. (3) and Eq. (4), respectively. The average rate of axial strain in the specimen is shown in Eq. (5), where ’$L_S$’ is the length of the specimen. Equation (6) gives the engineering strain ’$\epsilon _E$’ in the specimen. With the availability of the displacement and force data, the stresses on the input and output side of the specimen were determined. Equation (7) shows the force at the input side ($F_1$) and Eq. (8) shows the force at output side ($F_2$) of the specimen, where ’$A_B$’ is the cross-sectional area of the bar. The average stress in the specimen is then given by Eq. (9), where ’$A_S$’ is the cross-sectional area of the specimen.

To minimize radial inertia, the ratio of the specimen diameter to its length was maintained $\sim$ 3.5. The specimen (from each sequence) was mounted in the test section with facet plate facing the incident side and the distal plate facing the transmission side as shown in Fig. 5. The free surfaces of the specimen were lubricated with grease to reduce the interfacial friction. The diameters of the incident and transmission bars were equal to 12.5 mm. To investigate the effect of high strain rates on the stress-strain response of each sequence, compression pressures of 10 bar and 30 bar were chosen (loading section setting). The striking velocity ’$V_0$’ was noted during each trial, along with the strain and time data. The experiments were repeated for three specimens from each of the sequences. From the data, the strain-time plots and true stress-strain plots were obtained. The shock energy absorption for the different sequences was determined. The cross-sections of the tested specimens were inspected using optical microscopy (make: Olympus BX53M) and scanning electron microscopy (make: Zeiss EVO). A regression analysis was carried out between the Transmission Loss displayed by the sequences in our previous work^3^, and the shock energy absorption by the respective sequences at the two strain rates. To conduct the regression analysis, the MINITAB^®^ software was used.

## Results and discussion

### Response of the FMLs to the impedance tube experiments

**Figure 7:**
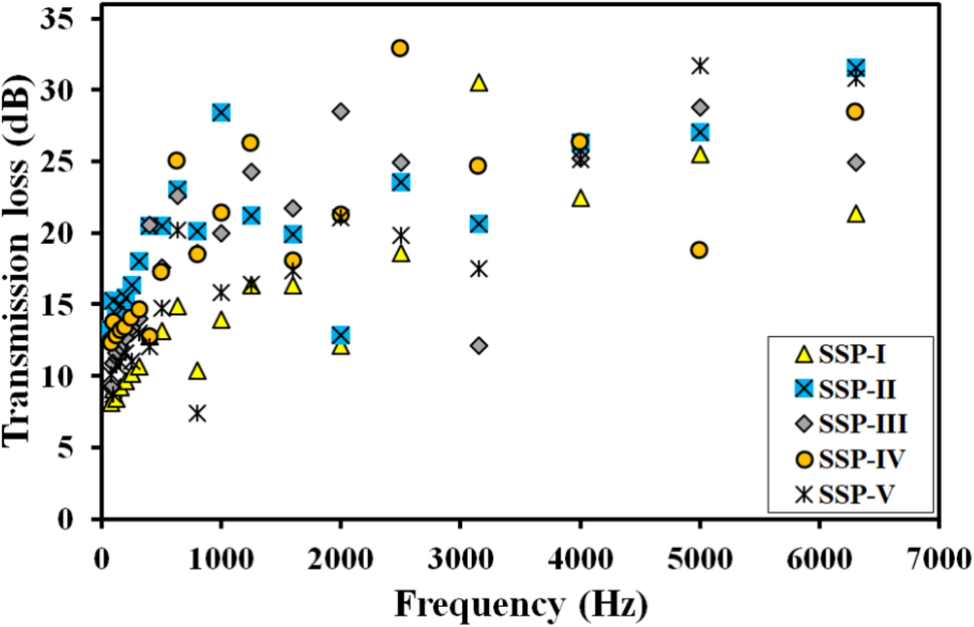
Transmission loss versus frequency using numerical model for sequences, SSP-I to SSP-V.

The results of the impedance tube experiments are shown in Fig. 7, with the variation of transmission loss with frequency for the different sequences. The sequences SSP-II and SRSP-IV demonstrated the largest values of transmission loss, exhibiting more peaks than the other sequences and a constant Transmission Loss value of over 15 dB between 500 Hz and 6.3 6300 Hz. Our recent paper has more information on the shock impedance matching of FMLs^29^. Table 3 shows the measured average transmission loss. The uncertainty for the transmission loss was estimated at 95% confidence interval, as shown in Eq. (10). SSP-II showed the highest Transmission Loss, followed by SSP-IV and SSP-III. The presence and location of the paperboard ply was critical in influencing the Transmission Loss characteristics of the sequences.

10 $$\begin{aligned} u_{m}=\sum _{i}^{N}\frac{\left( X_i-\bar{X}\right) ^2}{N\left( N-1\right) } \end{aligned}$$

**Table 3:** Transmission loss functions for the different stacking sequences.

Stacking sequence	Transmission loss (dB)			Laminate thickness (mm)
	$\bar{X}$	u$_{m}$	$\bar{X}$ ± 1.96u$_{m}$	
SSP-I	14.33	1.34	14.33 ± 2.63	3.43
SSP-II	20.17	1.03	20.17 ± 2.02	3.74
SRSP-III	18.68	1.37	18.68 ± 2.69	3.50
SSP-IV	19.29	1.39	19.29 ± 2.72	3.52
SSP-V	16.31	1.34	16.31 ± 2.63	2.84

### Response of the FMLs to SHPB experiments

**Figure 8:**
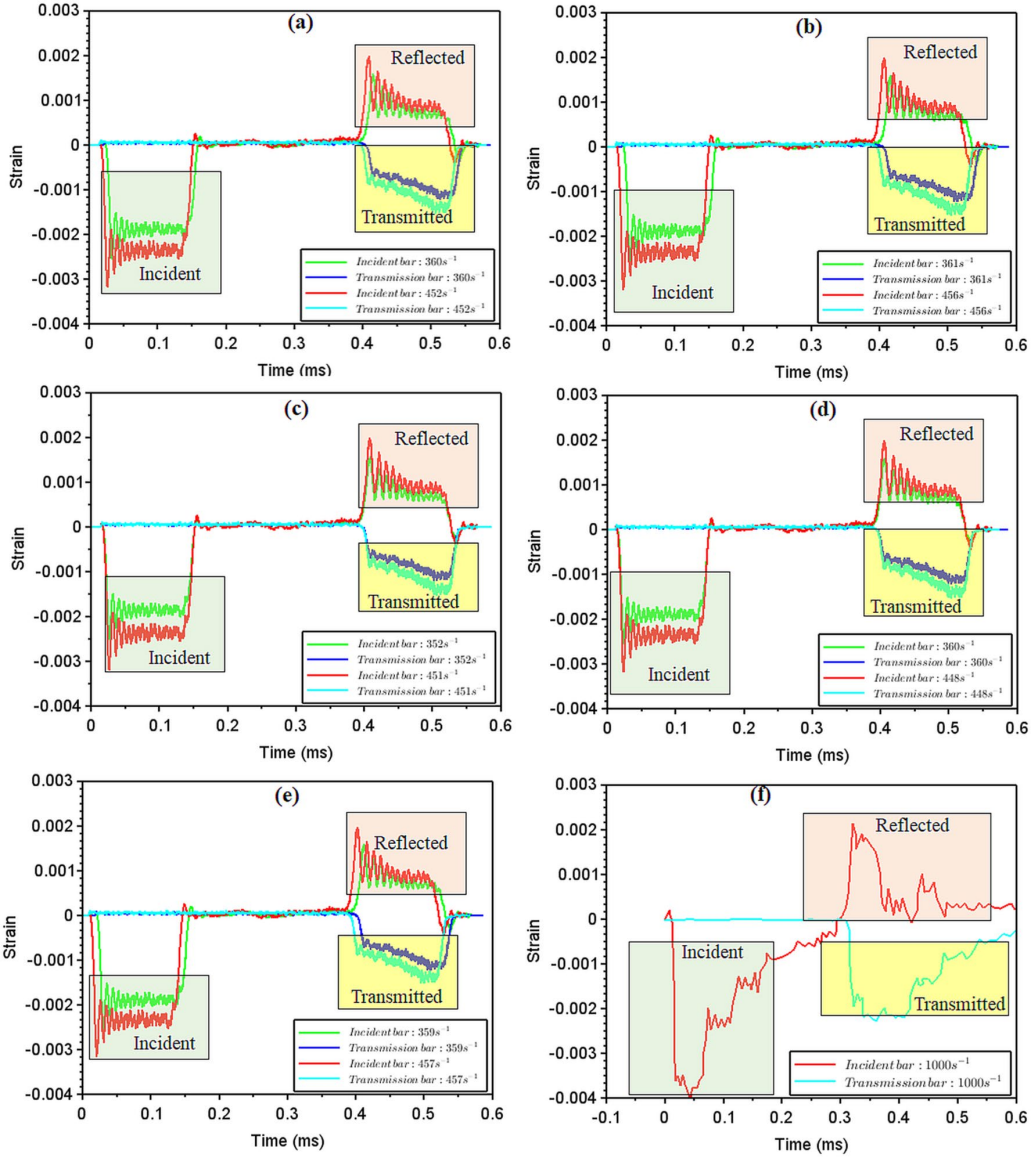
Strain–time plots for the different sequences comparing the incident, reflected and transmitted waves (**a**) SSP-I, (**b**) SSP-II, (**c**) SSP-III, (**d**) SSP-IV, (**e**) SSP-V, (**f**) AA 7449-T7651^57^.

The strain-time data for the different sequences have been plotted as shown in Fig. 8. For each sequence, the incident, reflected, and transmission strain values have been indicated. As the incident wave strikes the surface of the specimen, a portion gets transmitted through the specimen into the transmission bar, hence the strain amplitude is lower for the transmission side as measured by the strain gages; Another portion of the incident wave gets reflected into the incident bar, the sign of the strain curves is inverted owing to the reversal in the direction of shock travel and expectedly weaker with lower strain amplitude. As the striker velocity is increased (by increasing pressure on the pressure cylinder), the overall amplitude of the strain pulses (in incident, reflected and transmitted signals) is seen to increase, and this trend is seen throughout the sequences. The strain-time histories were compared with the results of high strain rate testing of AA7449-T7651 carried out by Mylonas et al^57^, displayed in Fig. 8f. Evidently, the strain rate used is 1000 s$^{-1}$, leading to the spike in the amplitude of the strains measured for the incident, transmitted and reflected compression waves.

**Figure 9:**
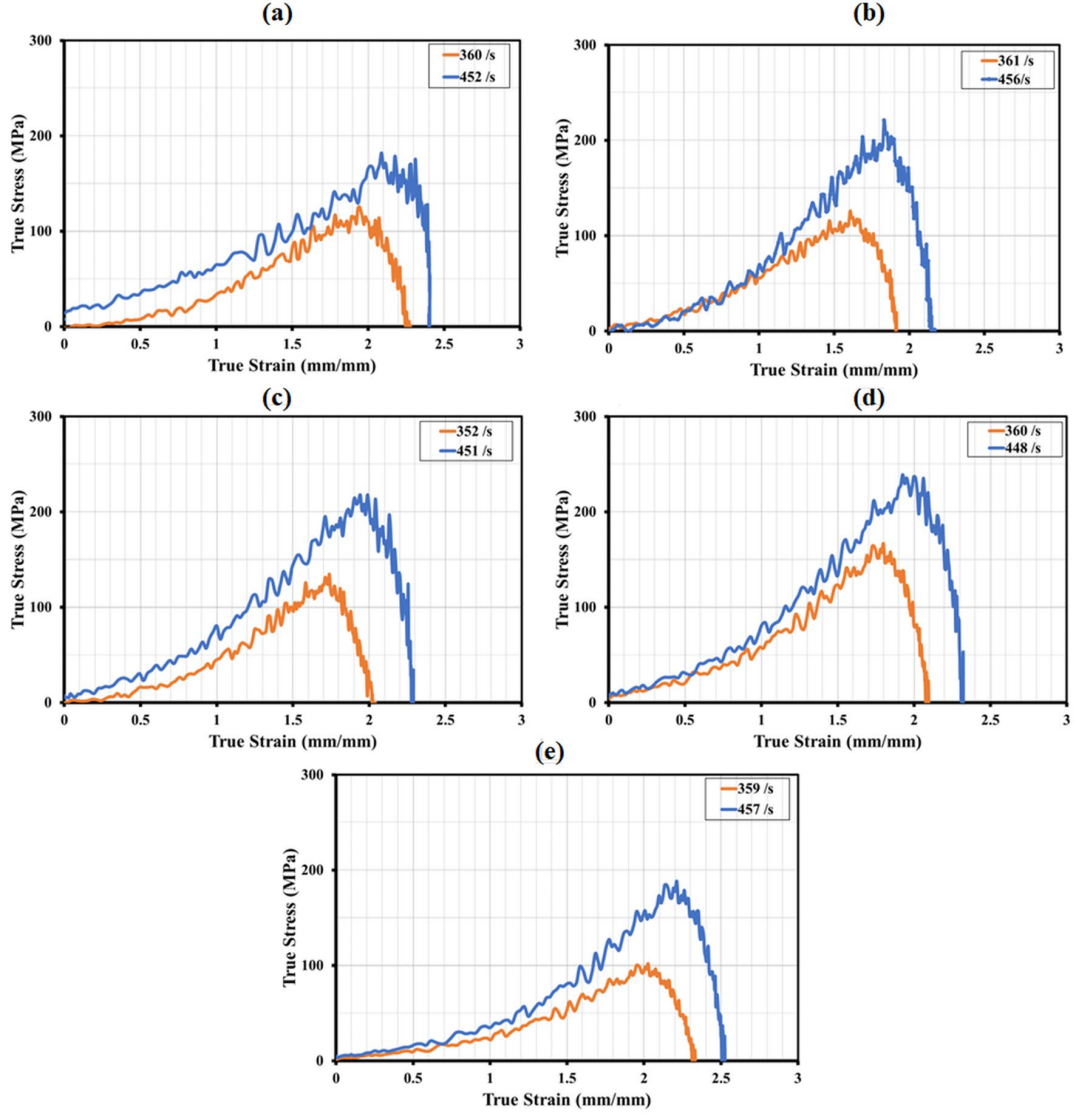
True stress–strain curves for the configurations (**a**) SSP-I, (**b**) SSP-II, (**c**) SSP-III, (**d**) SSP-IV, (**e**) SSP-V.

The true stress and strains were calculated using Eqs. (9 and 6) respectively^58–60^, the true stress–strain variation for the different sequences have been plotted in Fig. 9. It is evident that the stress-strain curves cannot be used to compute the elasticity modulus in the SHPB tests owing to the high strain rates, which disrupt the equilibrium conditions required for the test volume^57,61,62^. In all the sequences, the strain hardening effect was observed on increasing the strain rates. Strain hardening was observed in the sequences between 1.8 and 2.4 mm/mm. As AA6061-T6 is strain-hardenable, the facet and distal layers are where strain hardening occurs most frequently. The variation in the dislocation–dislocation interactions of the other constituent layers—aramid, UHMWPE and paperboard plies in the individual laminates may be responsible for the variation in the strain hardening for the various sequences. Thus, it can be surmised that arrangement of the constituent plies influences the response of the different sequences, and the acoustic impedance matching plays an important role in the transmittance of the compressive stresses from ply-to-ply^63–65^. The shock energy absorbed per unit volume was computed for all the sequences, the details have been shown in Table 4. At the higher strain rate of $460\, \textrm{s}^{-1}$, the highest shock energy absorption was shown by SSP-IV, followed by SSP-II, and SSP-III. When the strain rate was increased from 350 to 460 $s^{-1}$ , the highest increase in the strain energy absorption was shown by SSP-IV (76.2%), followed by SSP-II (68.7%), SSP-III (68.4%), SSP-V (68.1%) and SSP-I (65.9%). Shock energy absorption depends on the shock attenuation capability of the individual plies in the respective laminates. Coupled with the fact that the extent of strain hardening varies across the sequences due to the ply arrangement, the sequences with least shock transmission response, contribute to maximum shock energy absorption. Hence, among all the sequences, SSP-IV, SSP-II and SSP-III showed the best stress-strain response, strain hardening and shock energy absorption at high strain rates. The presence of the low acoustic impedance material, paperboard/epoxy ply as an intermediate layer in these sequences has positively contributed to the improved performance. The absence of AA6061-T6 faceplate in SRSP-V has led to a pronounced reduction in the shock energy absorption, although it was the lightest arrangement among all the sequence.

**Table 4:** Details of shock energy absorbed at different strain rates.

Configuration	Shock energy $(MJ/m^3)$ absorbed at		Increase (%)
	$$\sim $$ 350 s$$^{-1} $$	$$\sim $$ 460 s$$^{-1} $$	
SSP-I	14.1	23.4	65.96
SSP-II	16.3	27.5	68.71
SSP-III	15.2	25.6	68.42
SSP-IV	16.8	29.6	76.19
SSP-V	13.8	23.2	68.12

### Optical and SEM analysis of the specimens subjected to SHPB experiments

**Figure 10:**
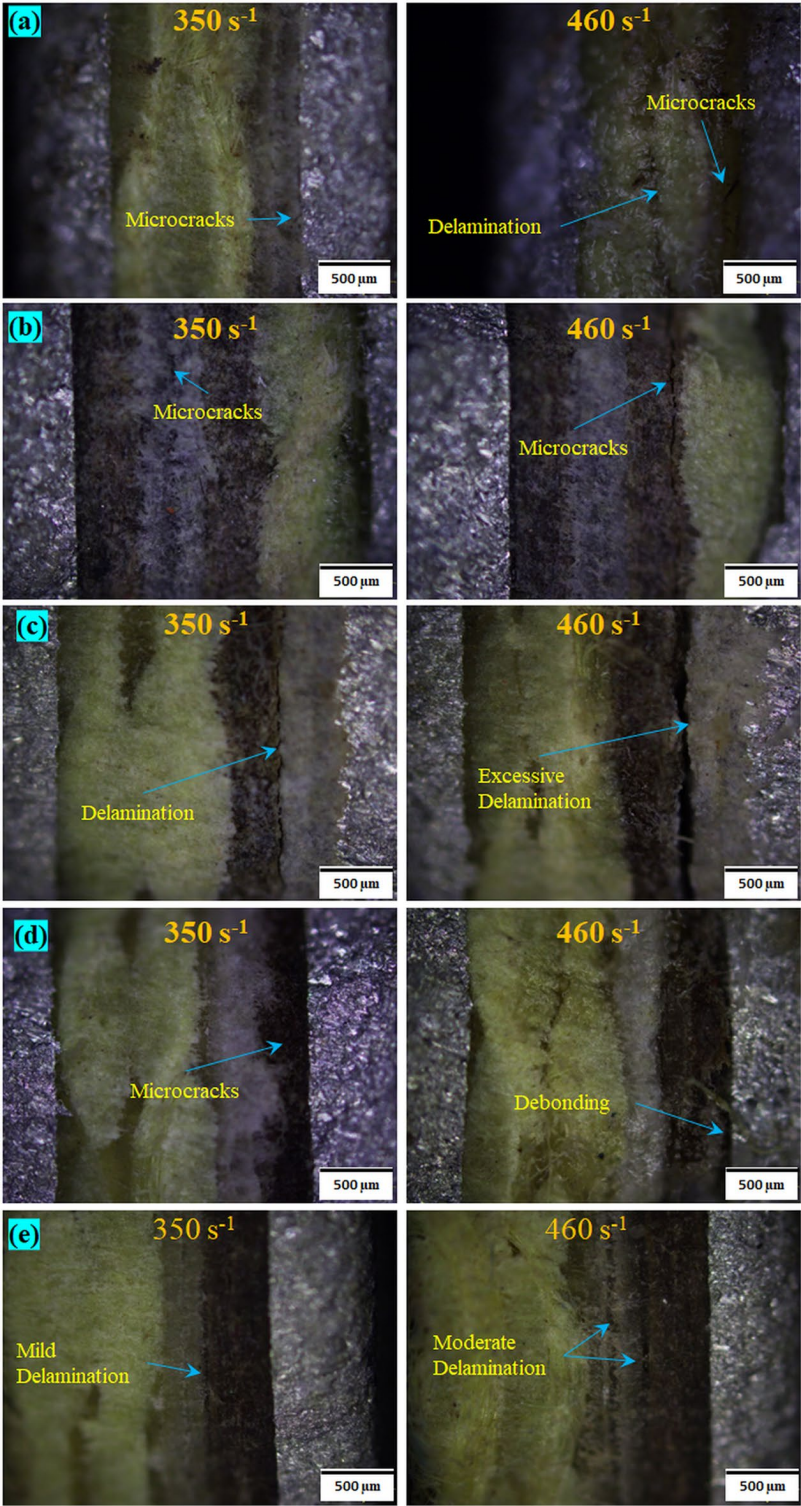
Optical micrographs of specimens subjected to SHPB tests at different strain rates (**a**) SSP-I, (**b**) SSP-II, (**c**) SSP-III, (**d**) SSP-IV, (**e**) SSP-V.

**Figure 11:**
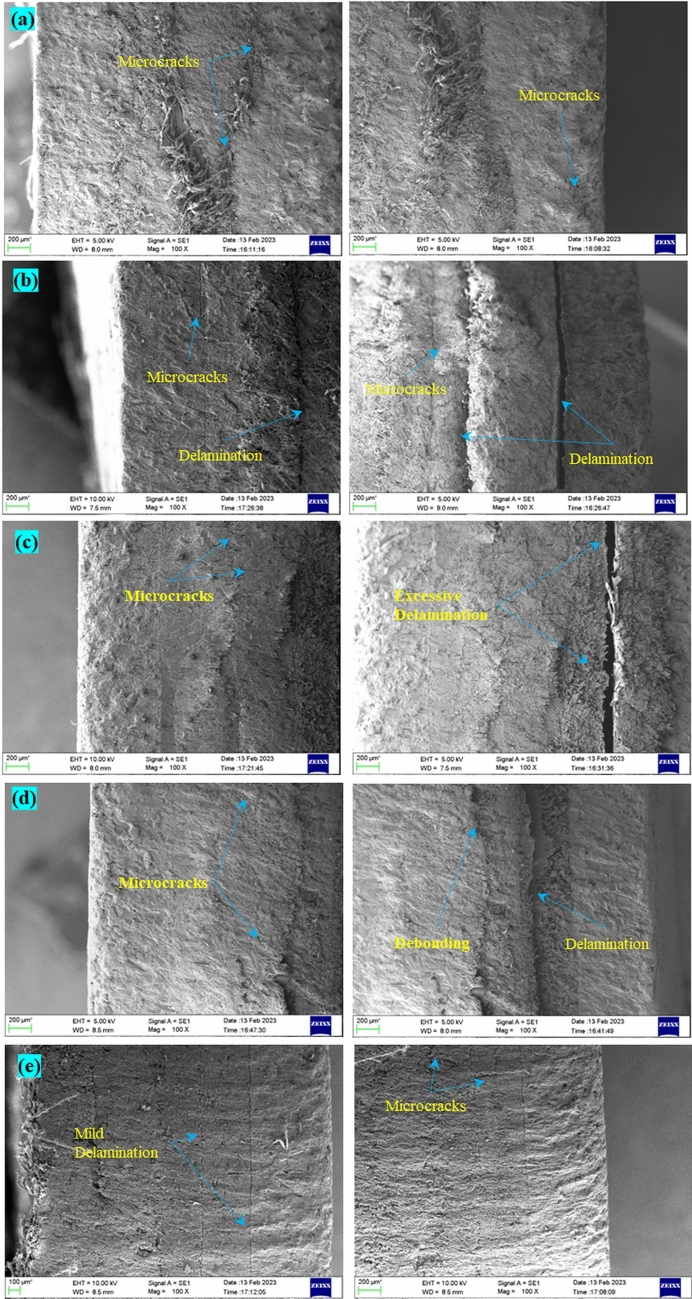
Scanning electron micrographs of some specimens subjected to SHPB tests at 450 s$^{-1}$ (**a**) SSP-I, (**b**) SSP-II, (**c**) SSP-III, (**d**) SSP-IV, (**e**) SSP-V.

The optical micrographs of the samples put through SHPB tests are shown in Fig. 10. The sequences SSP-I, SSP-II, and SSP-IV displayed microcracks in the penultimate layers at the lower strain rate, whereas SSP-III and SSP-V displayed delamination between the paperboard and UHMWPE layers. At the higher strain rate of 460 s$^{-1}$, the plies were subjected to aggravated failures. SSP-I showed many microcracks in the penultimate layer, and delamination between the 2nd and 3rd plies of aramid. SSP-II showed development of microcracks in the paperboard layer. SSP-III showed an aggravated delamination and separation between the paperboard and UHMWPE layers. SSP-IV showed a debonding between the paperboard and AA6061 layers. In SSP-V, the mild delamination at lower strain rate transformed to moderate delamination at the higher strain rate, attributed to the absence of a high shock impedance layer (AA6061) faceplate. The scanning electron micrographs of the SHPB tested specimens (at 450 s$^{-1}$) are shown in Fig. 11. All of the sequences displayed microcracks in the intermediate plies. SRSP-II and SRSP-III displayed severe delamination between the paperboard and UHMWPE layers. SRSP-IV showed a mild delamination between the UHMWPE and paperboard. In SRSP-V, the mild to moderate delaminations were observed in the intermediate plies in addition to the microcracks.

### Regression analysis between shock impedance matching and shock energy absorption

**Table 5:** Regression analysis table for shock energy absorption at 350 s$^{-1}$ and transmission loss: coefficients.

Term	Coefficient	SE coefficient	T-value	P-value
Constant	6.121	2.860	2.14	0.122
Transmission loss	0.5116	0.1594	3.21	0.049

**Table 6:** Regression analysis table for shock energy absorption at 350 s$^{-1}$ and transmission loss: model summary.

S	R-Sq (%)	R-Sq(adj) (%)
0.721946	77.4	69.9

**Figure 12:**
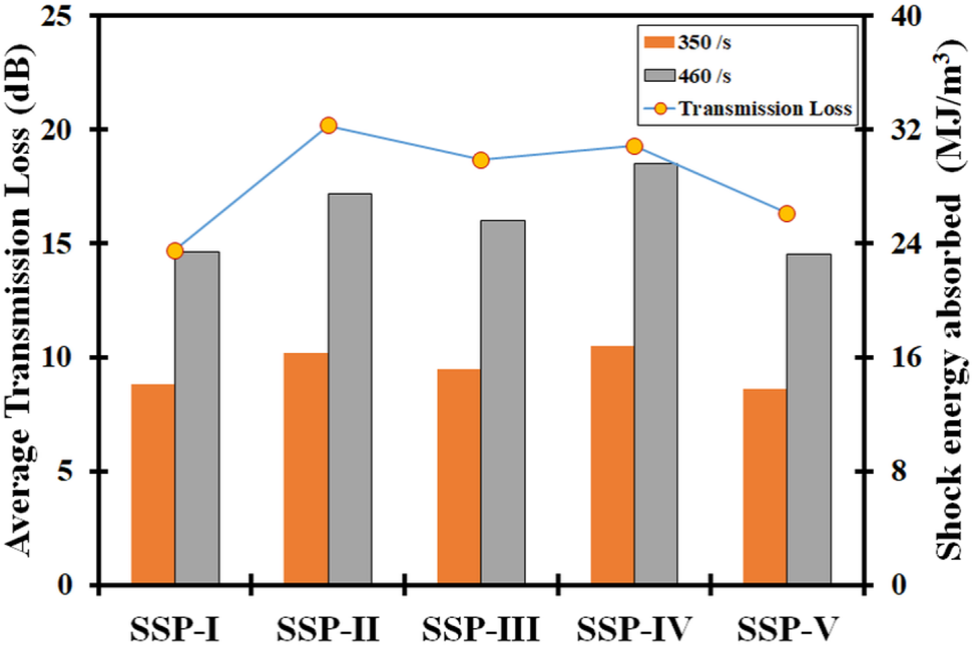
Comparison of transmission loss of the sequences with the shock energy absorption^29^.

11 $$\begin{aligned} {SE}_{abs,1}=6.12+0.512\times {TL} \end{aligned}$$

**Table 7:** Analysis of variance table for shock energy absorption at 350 s$^{-1}$ and transmission loss: coefficients.

Source	DoF	Adj. SS	Adj. MS	F-value	P-value
Regression	1	5.3684	5.3684	10.30	0.049
Error	3	1.5636	0.5212		
Total	4	6.9320			

**Figure 13:**
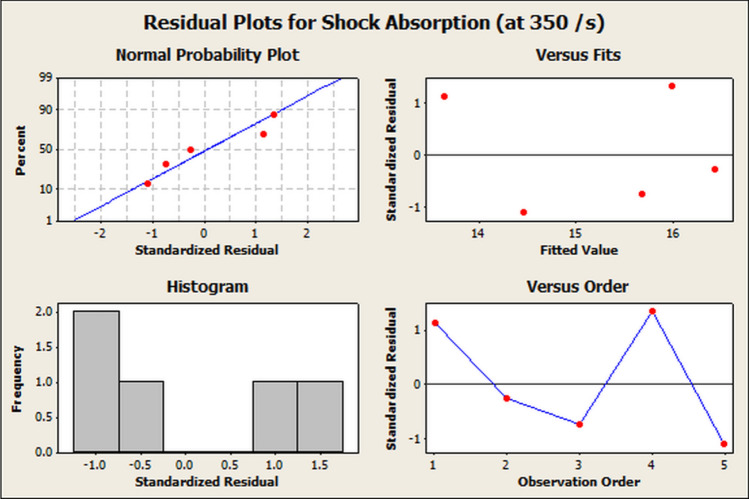
Residual plots for shock energy absorption at 350 s$^{-1}$ and transmission loss.

**Figure 14:**
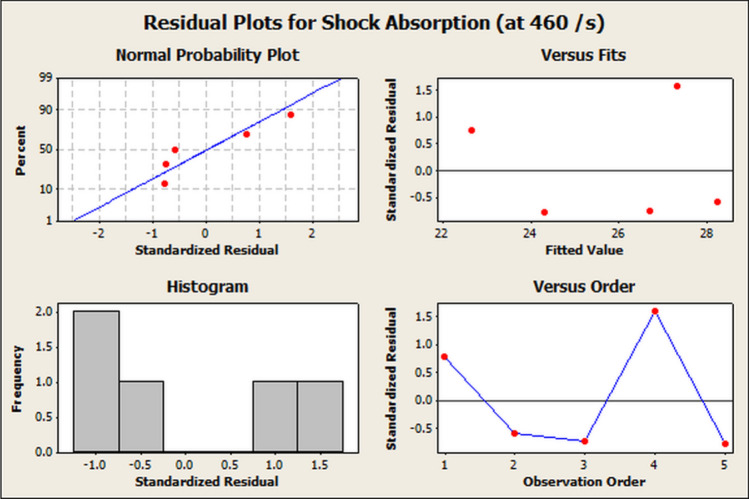
Residual plots for shock energy absorption at 460 s$^{-1}$ and transmission loss.

The regression equation between the transmission loss and the shock energy absorption (${SE}_{abs,1}$) is shown in Eq. (11). The regression analysis tables for the shock energy absorption at 350 s$^{-1}$ are shown in Tables 5 and 6, respectively.

The comparison of the Transmission Loss and the shock energy absorption data is shown in Fig. 12. The shock impedance mismatch introduced by the varied arrangement of the core layers plays an important role in shock attenuation in the sequences. SSP-II displaying a high Transmission Loss was found to display the second highest shock energy absorption at both the tested high strain rates, while SSP-IV showed the second highest Transmission Loss with the highest shock energy absorption among the sequences. SSP-III showed the third highest Transmission Loss and shock energy absorption among the sequences. The remaining two sequences, SSP-I and SSP-II although displayed lower Transmission Losses and lower shock energy absorption as compared to the other sequences, there was a close agreement between the Transmission Loss values and the shock absorption at the two strain rates too.

The ANOVA is shown in Table 7. A significance level of $\alpha \sim 0.05$ was taken during the analysis. The degree of freedom (DoF), adjusted sum of squares (Adj. SS), adjusted mean squares (Adj. MS), the *F*-Value and *P*-Value indicate the level of significance of the parameters. It was seen from Table 7 that ’P-Value’ < $\alpha$, which indicated the high level of significance for the study. The corresponding residual plots are shown in Fig. 13. The regression equation between the Transmission Loss and the shock energy absorption (${SE}_{abs,2}$) is shown in Eq. (12). The Regression analysis tables for the shock energy absorption at 460 s$^{-1}$ are shown in Tables 8 and 9 respectively. The ANOVA is shown in Table 10. It was observed from Table 10 that ’P-value’ < $\alpha$, which indicated the high level of significance for the shock energy absorption and Transmission Loss. The corresponding residual plots are shown in Fig. 14.

12 $$\begin{aligned} {SE}_{abs,2}=7.744 + 1.02\times {TL} \end{aligned}$$

**Table 8:** Regression analysis table for shock energy absorption at 460 s$^{-1}$ and transmission loss : coefficients.

Term	Coefficient	SE coefficient	T-value	P-value
Constant	7.744	6.739	1.15	0.334
Transmission loss	1.0164	0.3756	2.71	0.049

**Table 9:** Regression analysis table for shock energy absorption at 460 s$^{-1}$ and transmission loss: model summary.

S	R-Sq (%)	R-Sq(adj) (%)
1.70124	70.9	61.2

**Table 10:** Analysis of variance table for shock energy absorption at 460 s$^{-1}$ and transmission loss.

Source	DoF	Adj. SS	Adj. MS	F-value	P-value
Regression	1	21.189	21.189	7.32	0.049
Error	3	8.683	2.894		
Total	4	29.872			

## Conclusion

Five configurations of fiber-metal laminates comprising AA6061-T6 skins, aramid, UHMWPE, and paperboard layers as core layers were experimentally characterized for Transmission Loss response on an impedance tube and high strain rate response on split hopkinson pressure bar. Based on the behaviour of the different sequences, the following conclusions were made:

- The shock impedance tuning influences the Transmission Loss functions of the sequences. Among the five sequences, SSP-II (20.17 ± 2.63 dB), SSP-IV (19.29 ± 2.72 dB), and SSP-III (18.68 ± 2.69 dB) showed the highest values of the average Transmission Loss. Thus, the location of the low impedance paperboard ply in the stackup, minimized the transmitted energy (acoustic) in the sequences.
- The dynamic stress-strain curves display a marked rise at higher strain rates. The failure strains were found to reduce with increase in the strain rate. The SSPs offered enhanced capability to absorb shock energies.
- The sequence SSP-IV followed by SSP-II and SSP-III displayed the highest energy absorption at the high strain rates 350 s$^{-1}$ to 460 s$^{-1}$. The addition of a low shock impedance ply as an intermediate ply assists in improving the strain rate sensitivity of fiber-metal laminates. Absence of metallic ply as facing layer severely affected the response of SSP-V, which backs the role of metals and alloys as prime facing materials in hybrid laminates.
- The primary failure modes in the laminates comprised micro-cracks, debonding and delamination among plies. The delamination was predominant at the paperboard ply interface, as seen in the sequences SSP-II, SSP-III, SSP-IV and SSP-V.
- The regression analysis between the shock energy absorption at the two high strain rates and the transmission loss displayed a high level of significance ($<\alpha$=0.05). The regression coefficients were obtained as R$^2$=0.77 at 350 s$^{-1}$ and R$^2$=0.71 at 460 s$^{-1}$. The Shock energy absorption was shown as a function of transmission loss for the respective strain rates.

Future advancements in manufacturing methods, like automated layup methods and additive manufacturing, may significantly improve the production of FMLs. These developments may reduce costs, increase manufacturing effectiveness, and make it possible to create intricate FML structures with specific features. The reliability and safety of aircraft structures can be greatly increased through multifunctional integration like the ones covered in this research.

## Data Availability

Data will be made available on reasonable request by the corresponding author.

## List of symbols

$$ {SE}_{abs,1}$$: Shock energy absorption at 350 s$$^{-1}$$
$$ {SE}_{abs,2}$$: Shock energy absorption at 460 s$$^{-1}$$
$\bar{X}$: Arithmetic mean of the transmission loss values for the corresponding sequence (dB)
$$\dot{\epsilon }$$: Strain rate (1/s)
$\epsilon _E$: Engineering strain (mm/mm)
$\epsilon _I$: Incident strain (mm/mm)
$\epsilon _R$: Reflected strain (mm/mm)
$\epsilon _T$: Transmitted strain (mm/mm)
$\rho _{m}$: Mass density of the bar material (kg$_{\textrm{m}^3}$)
$$\tau _c$$: Transmission coefficient
$A_B$: Cross-sectional area of the bar (m$^2$)
$A_S$: Cross-sectional area of the specimen (m$^2$)
$c_B$: Elastic wave velocity through the bar (m/s)
$E_m$: Young’s modulus of the elastic material of the bar (GPa)
$F_1$: Force on left side of specimen (N)
$F_2$: Force on right side of specimen (N)
$I_{inc}$: Incident energy (kJ)
$I_{tran}$: Transmitted energy (kJ)
$L_s$: Specimen length (mm)
*TL*: Transmission loss
$u_{m}$: Uncertainty of measurement in transmission loss
$V_1$: Left face velocity (m/s)
$V_2$: Right face velocity (m/s)
$X_i$: Transmission loss (dB) for ith reading
N: Number of values of transmission loss readings for the sequence
